# Removal mechanisms and kinetics of trace tetracycline by two types of activated sludge treating freshwater sewage and saline sewage

**DOI:** 10.1007/s11356-012-1213-5

**Published:** 2012-10-02

**Authors:** Bing Li, Tong Zhang

**Affiliations:** Environmental Biotechnology Laboratory, Department of Civil Engineering, The University of Hong Kong, Pokfulam Road, Hong Kong, SAR China

**Keywords:** Trace antibiotics, Activated sludge, Removal mechanisms, Sewage, Adsorption kinetics, Adsorption isotherm

## Abstract

Understanding the removal mechanisms and kinetics of trace tetracycline by activated sludge is critical to both evaluation of tetracycline elimination in sewage treatment plants and risk assessment/management of tetracycline released to soil environment due to the application of biosolids as fertilizer. Adsorption is found to be the primary removal mechanism while biodegradation, volatilization, and hydrolysis can be ignored in this study. Adsorption kinetics was well described by pseudo-second-order model. Faster adsorption rate (*k*_2_ = 2.04 × 10^−2^ g min^−1^ μg^−1^) and greater adsorption capacity (*q*_e_ = 38.8 μg g^−1^) were found in activated sludge treating freshwater sewage. Different adsorption rate and adsorption capacity resulted from chemical properties of sewage matrix rather than activated sludge surface characteristics. The decrease of tetracycline adsorption in saline sewage was mainly due to Mg^2+^ which significantly reduced adsorption distribution coefficient (*K*_d_) from 12,990 ± 260 to 4,690 ± 180 L kg^−1^. Species-specific adsorption distribution coefficients followed the order of $K_{\mathrm{d}}^{{ + 00}} \gg K_{\mathrm{d}}^{{ + - 0}} > K_{\mathrm{d}}^{{ + - - }}$. Contribution of zwitterionic tetracycline to the overall adsorption was >90 % in the actual pH range in aeration tank. Adsorption of tetracycline in a wide range of temperature (10 to 35 °C) followed the Freundlich adsorption isotherm well.

**Electronic supplementary material:**

The online version of this article (doi:10.1007/s11356-012-1213-5) contains supplementary material, which is available to authorized users.

## Introduction

In recent years, the occurrence and fate of antibiotics in the environment has drawn great attention of researchers all over the world (Kümmerer 2001; Xiao et al. 2008). Although the antibiotic residues in the environment are at the subinhibitory concentrations, they are still considered to be emerging pollutants because antibiotics may result in the development/maintenance/transfer/spread of antibiotic-resistant bacteria and antibiotic-resistant genes in the long term (Kim et al. 2005; Knapp et al. 2008; Martínez 2008).

Tetracyclines, which ranked the second in production and usage among all antibiotic classes worldwide, are widely used as human and veterinary medicine as well as growth promoter (Gu and Karthikeyan 2005). However, tetracyclines are poorly metabolized or absorbed in the digestive tract and 50–80 % is excreted through feces and urine as unchanged form (Sarmah et al. 2006). For the human-use portion, wastewater treatment plants (WWTPs) are one of the dominant sources of tetracyclines which are released into the environment through effluent and biosolids as fertilizer because WWTPs cannot remove tetracyclines completely (Miao et al. 2004). For the animal-use portion, tetracyclines enter into the environment mainly through application of manure and waste lagoon water to fields as fertilizer (Boxall et al. 2004). Consequently, tetracyclines have been frequently detected in the environment, including effluent and sludge from WWTPs (Li et al. 2009; Miao et al. 2004; Spongberg and Witter 2008), surface water (Kim and Carlson 2007), sediment (Kim and Carlson 2007), and soils (Aga et al. 2005) around the world.

To date, although tetracyclines were found to be eliminated to some degree in the activated sludge process with the removal efficiencies of 11.6 % (Spongberg and Witter 2008) to 85.4 % (Batt et al. 2007), less attention was paid to their removal mechanisms (biodegradation, adsorption, volatilization, or hydrolysis) at environmentally relevant concentrations and the corresponding systematic studies were very limited. Understanding the removal of tetracycline by activated sludge is not only critical to the evaluation of tetracycline elimination in WWTPs but also to the mass load prediction and risk assessment/management of tetracycline released to the soil environment since biosolids derived from WWTPs are widely applied to fields as fertilizer (Monteiro and Boxall 2009). Adsorption is an important process controlling the transport and fate of tetracyclines in the environment. Recent studies on adsorption of tetracyclines mainly focused on using isolated clays (Avisar et al. 2010; Chang et al. 2009), aluminum hydrous oxide (Gu and Karthikeyan 2005), soils (Sassman and Lee 2005; Wan et al. 2010), sediment (Xu and Li 2010), sand (Zhang et al. 2012), humic substances/clay–humic complexes (Pils and Laird 2007; Sun et al. 2010), and carbon nanotubes (Ji et al. 2010) as adsorbents. However, the removal behavior of tetracyclines obtained based on the above studies cannot be applied directly to activated sludge (AS) due to the vast difference between AS and those adsorbents as well as the solution chemistry conditions. In addition, the initial tetracycline concentrations for most previous studies were several orders of magnitude higher than their environmentally relevant concentrations, usually ranging from a few to hundreds milligrams per liter level. This might result in significant bias when predicting the removal behavior at environmentally relevant concentration levels. Moreover, the situation in Hong Kong is much more special and unique as some WWTPs (e.g., Shatin WWTP) treat saline sewage resulting from the practice of seawater toilet flushing. The constituents of the saline sewage are much more complicated than the freshwater sewage, and the different aqueous solution chemistry properties might lead to remarkably different removal behavior in AS process. To our knowledge, this is the first study to systematically examine the elimination of tetracycline, a principal member of tetracyclines, by two types of AS treating freshwater sewage and saline sewage, respectively, at environmentally relevant concentrations. Particular emphasis was placed on investigating (1) the removal mechanisms (biodegradation, adsorption, volatilization, and hydrolysis) for tetracycline, (2) adsorption kinetics and isotherms of tetracycline on AS, (3) the effect of ion species and ion concentrations on adsorption, and (4) the impact of pH and calculation of species-specific adsorption distribution coefficients.

## Materials and methods

### Chemicals and standards 

The standard of tetracycline (purity >98 %) was purchased from Sigma-Aldrich. LC-MS grade acetonitrile was purchased from Fisher Scientific UK Limited. Ultrapure water was prepared using Easypure® UV/UF compact reagent-grade water system (Barnstead, Boston, USA). The following chemicals were all purer than analytical grade: formic acid (99 %) from Fluka, sodium hydroxide (≥97 %) from BDH, VWR International Ltd., hydrochloric acid (37 %) from E. Merck, rhodamine B (~95 %) and hexadecane (≥99 %) from Sigma, individual standard solutions (1 g L^−1^) of sodium, calcium, magnesium, chloride, and sulfate ions from Alltech Associates, Inc. (USA). Cellulose nitrate membrane (0.2 μm) was purchased from MFS® (Japan).

### Removal of tetracycline in activated sludge process

The saline sewage, freshwater sewage, and AS were collected from the aeration tanks (aerobic stage) of two local wastewater plants, i.e., Shatin and Stanley WWTPs, in August and September 2010, respectively. Table S1 (see Electronic supplementary material) shows the details of the two WWTPs. The major removal mechanisms for antibiotics in activated sludge process are considered to be biodegradation, adsorption, volatilization (due to aeration), and hydrolysis (Kim and Aga 2007; Pérez et al. 2005). In order to distinguish the primary removal mechanisms for tetracycline, batch test utilizing seven 2-L glass beakers with 1 L mixed liquor was run simultaneously at 25 ± 1 °C for 24 h. The detailed information on the experiment design was summarized in Table S2 and Section S 2.2 (see Electronic supplementary material).

### Characterization of sewage 

The sewages were first filtered using a 0.45-μm cellulose nitrate membrane at the sampling site, and then 50 mL filtrate was kept in an ice box and transported to the laboratory for the following analyses. The dissolved organic carbon (DOC) was measured using a total organic carbon analyzer (TOC-V_CPH,_ Shimadzu, Japan). The cations (Na^+^, Mg^2+^, and Ca^2+^) and anions (Cl^−^ and SO_4_^2−^) were analyzed by an ion chromatograph (COD-6A, Shimadzu, Japan), and the salinity was detected by a conductivity meter (Model 135A, ORION, USA).

### Characterization of AS

The characterization tests of two types of AS, i.e., zeta potential, particle size distribution, specific surface area, and relative hydrophobicity (RH), were conducted in duplicate within 10 h after sample collection.

#### Zeta potential

The original sludge samples were first mixed thoroughly by a vortex mixer for 5 min for homogenization. Then, the supernatant was sampled after a 10-min settling for zeta potential measurement using Delsa™ Nano Series Zeta Potential Analyzers (Delsa™ Nano C, Beckman, USA) (Chang et al. 2001).

#### Particle size distribution

The size distribution of AS was determined by a laser diffraction particle size analyzer (LS™ 13 320, Beckman, USA). Both the mean and median values were utilized to characterize sludge size.

#### Specific surface area

The specific surface area of AS was determined using the rhodamine B adsorption method which assumes that the adsorption isotherm fits the Langmuir model (Smith and Coackley 1983; Sørensen and Wakeman 1996). In this study, the adsorption isotherm test was conducted at 25 °C with the initial rhodamine B concentration of 0.5 to 75 mg L^−1^. The AS concentration was 0.5 g SS L^−1^, and the equilibrium time was 48 h. Rhodamine B was measured using a spectrophotometer (HACH, DR/2400) at the wavelength of 553 nm. Thus, the specific surface area can be calculated according to Eq. (1) (Smith and Coackley 1983):

1 $$ S = {{q}_{{\max }}} \times N \times A $$

where *S* is the specific surface area of the AS (in square meters per gram), *q*_max_ is the monolayer saturation adsorption capacity (in moles per gram) obtained from the Langmuir model fitting, *N* is Avogadro’s number (6.023 × 10^23^ molecules mol^−1^), and *A* is the area occupied by a single rhodamine B molecule (1.95 × 10^−18^ m^2^ molecule^−1^) (Laurent et al. 2009).

#### Relative hydrophobicity

The RH was evaluated following the protocol of Wilén et al. (2003). Thirty milliliters of AS mixed liquor was agitated uniformly with 15 mL hexadecane for 10 min in a separatory funnel. After a 30-min settling, the two phases were completely separated and the aqueous phase was transferred into another beaker. The RH was calculated using Eq. (2):

2 $$ {\mathrm{RH}}\left( \% \right) = \left( {1 - \frac{{{\mathrm{MLS}}{{\mathrm{S}}_{\mathrm{e}}}}}{{{\mathrm{MLS}}{{\mathrm{S}}_{\mathrm{i}}}}}} \right) \times 100 $$

where MLSS_i_ and MLSS_e_ are the AS concentrations in the aqueous phase before and after emulsification.

### Batch adsorption studies 

All batch adsorption experiments were conducted in 100-mL conical flasks with rubber stoppers which were shaken on an orbital shaking incubator at 125 rpm in the dark to avoid possible photolysis of tetracycline. Stock solutions (100 mg L^−1^) were freshly prepared daily by dissolving 10 mg tetracycline in 100 mL deionized water. About 20 L mixed liquor sampled from aeration tank was first settled by gravity for 30 min to concentrate the sludge and then the supernatant was filtered by a 0.45-μm cellulose nitrate membrane. The obtained “filtrate” (15 L) was kept at 4 °C before being used in the following batch adsorption experiments. Sodium azide was added into the concentrated sludge (~5 L) to get a final concentration of 1 % (*w*/*v*) to inhibit the microbial activity by incubation for 48 h at room temperature (Batt et al. 2007). Then, 0.05 g sludge (MLSS) after the above treatment was collected by centrifugation at 4,000 rpm for 5 min and resuspended in 50 mL filtrate to achieve a final concentration of 2.5 g L^−1^. Unless otherwise specified, the pH of the above suspension was adjusted to 7.00 ± 0.05 with HCl or NaOH solutions and the initial tetracycline concentration was 100 μg L^−1^. The temperature was controlled at 25 ± 0.5 °C, and the equilibrium time of 24 h was chosen based on the adsorption kinetics study. Duplicate experiments were performed in parallel.

#### Adsorption kinetics

Four groups of experiments were conducted in the kinetic study: (I) Stanley AS in the freshwater sewage filtrate (FSF), (II) Shatin AS in the saline sewage filtrate (SSF), (III) Stanley AS in SSF, and (IV) Shatin AS in FSF. Groups (I) and (II) represented the actual adsorption conditions in the AS process of Stanley and Shatin WWTPs, respectively. Groups (III) and (IV) were used to distinguish the key factors (AS properties and/or the sewage matrix) which lead to the different adsorption behavior in these two WWTPs. A series of sealed conical flasks containing the above 50 mL AS slurry with initial TC concentrations of 100 μg L^−1^ were shaken continuously on an orbital shaking incubator (125 rpm) at 25 ± 0.5 °C in the dark. Blank samples without AS were kept in the same condition as the control. The conical flasks were taken out at different time intervals (2 min, 5 min, 10 min, 15 min, 30 min, 1 h, 2 h, 5 h, 10 h, 15 h, and 24 h) for subsequent tetracycline detection.

#### Effect of ion species/concentration

According to major ion species and concentrations detected in saline wastewater, NaCl, CaCl_2_, MgCl_2_, and Na_2_SO_4_ were all spiked into FSF to simulate the SSF, called as synthetic saline sewage (SSS). In addition, to distinguish the effects of different ions on tetracycline adsorption, the above salts were individually spiked into FSF at three concentration levels and the middle level was at their actual concentrations in SSF. All the experiments in this section used Shatin AS as the adsorbent. Blank samples without Shatin AS were used as the controls. The adsorption distribution coefficient *K*_d_, which was used to examine the effect of the ion species and concentration on adsorption, is defined as follows:

3 $$ {{K}_{\mathrm{d}}} = \frac{{{{q}_{\mathrm{e}}}}}{{{{C}_{\mathrm{e}}}}} = \frac{{\left( {{{C}_0} - {{C}_{\mathrm{e}}}} \right) \cdot V/M}}{{{{C}_{\mathrm{e}}}}} $$

where *q*_e_ (in micrograms per gram) is the amount of tetracycline adsorbed per gram sludge at equilibrium and *C*_e_ (in micrograms per liter) is the equilibrium aqueous tetracycline concentration.

#### Effects of pH

The suspension pHs were adjusted between 4.50 and 9.00 (0.50-unit increments) by adding small volumes of hydrochloric acid or sodium hydroxide solutions. Final pH values were measured at the equilibrium time of 24 h. Blank samples without AS were used as the controls.

#### Adsorption isotherms

Adsorption isotherms were obtained to assess tetracycline distributions between AS and aqueous phases as a function of the tetracycline concentration and temperature (Figueroa et al. 2004). The experiments were conducted at nine initial concentrations ranging from 1 to 100 μg L^−1^ under three temperatures, i.e., 10, 25, and 35 °C, respectively. Both the concentration and temperature were selected at the environmentally relevant levels. Blank samples without AS were used as the controls for each concentration.

### Ultraperformance liquid chromatography–tandem mass spectrometry analysis

All the samples were filtered via a 0.2-μm cellulose nitrate membrane which showed no adsorption of tetracycline (Li et al. 2009), kept in dark at 4 °C, and analyzed directly via Acquity™ ultraperformance liquid chromatography–tandem mass spectrometry (UPLC-MS/MS, Waters) within 24 h. UPLC-MS/MS was operated in the positive electrospray ionization multiple reaction monitoring (MRM) mode. The tetracycline concentrations were quantified using external calibration method, and the standards were prepared using the corresponding sewage filtrate to correct the matrix effect. The limit of quantification of tetracycline was 0.05 μg L^−1^. Sample pretreatment and mobile phase gradient were summarized in the Electronic supplementary material while other detailed information on MRM parameters, column, flow rate, and formic acid concentration were reported in the previous study (Li et al. 2009).

## Results and discussion

### Removal of tetracycline in activated sludge process

As shown in Fig. S1 (see Electronic supplementary material), tetracycline was found to be stable and no hydrolysis occurred during the testing period, and the elimination due to volatilization can be ignored based on the data of treatments III and IV. Thus, only biodegradation and adsorption might account for the removal of tetracycline. However, the strong similarity between treatment I profile and treatment II profile suggests that adsorption is the primary mechanism for tetracycline removal in both freshwater and saline sewage activated sludge systems while biodegradation can be completely ignored (Fig. S1). In addition, it should be noted that different adsorption rate and adsorption capacity were found in the two systems, and these will be discussed in detail in the following sections.

### Batch adsorption studies and characterization of AS and sewage

#### Adsorption kinetics

In general, three kinetic models, i.e., pseudo-first-order kinetics, pseudo-second-order kinetics, and Elovich model, were utilized to fit the adsorption data (Chang et al. 2009; Xu et al. 2009).

Pseudo-first-order kinetics can be expressed as the Lagergren’s rate equation:

4 $$ \log \left( {{{q}_{\mathrm{e}}} - {{q}_{\mathrm{t}}}} \right) = \log \left( {{{q}_{\mathrm{e}}}} \right) - \frac{{{{k}_1}}}{{2.303}}t $$

Pseudo-second-order kinetics can be written as the following equation:

5 $$ {{q}_{\mathrm{t}}} = \frac{{{{k}_2}q_{\mathrm{e}}^2t}}{{1 + {{k}_2}{{q}_{\mathrm{e}}}t}} $$

Equation (6) is the rearranged linear form:

6 $$ \frac{t}{{{{q}_{\mathrm{t}}}}} = \frac{1}{{{{k}_2}q_{\mathrm{e}}^2}} + \frac{1}{{{{q}_{\mathrm{e}}}}}t $$

Elovich model is expressed as

7 $$ {{q}_{\mathrm{t}}} = a\ln (t) + b $$

where *k*_1_ is the pseudo-first-order rate constant, *k*_2_ is the pseudo-second-order rate constant, and *a* and *b* are the Elovich model constants.

**Fig. 1:**
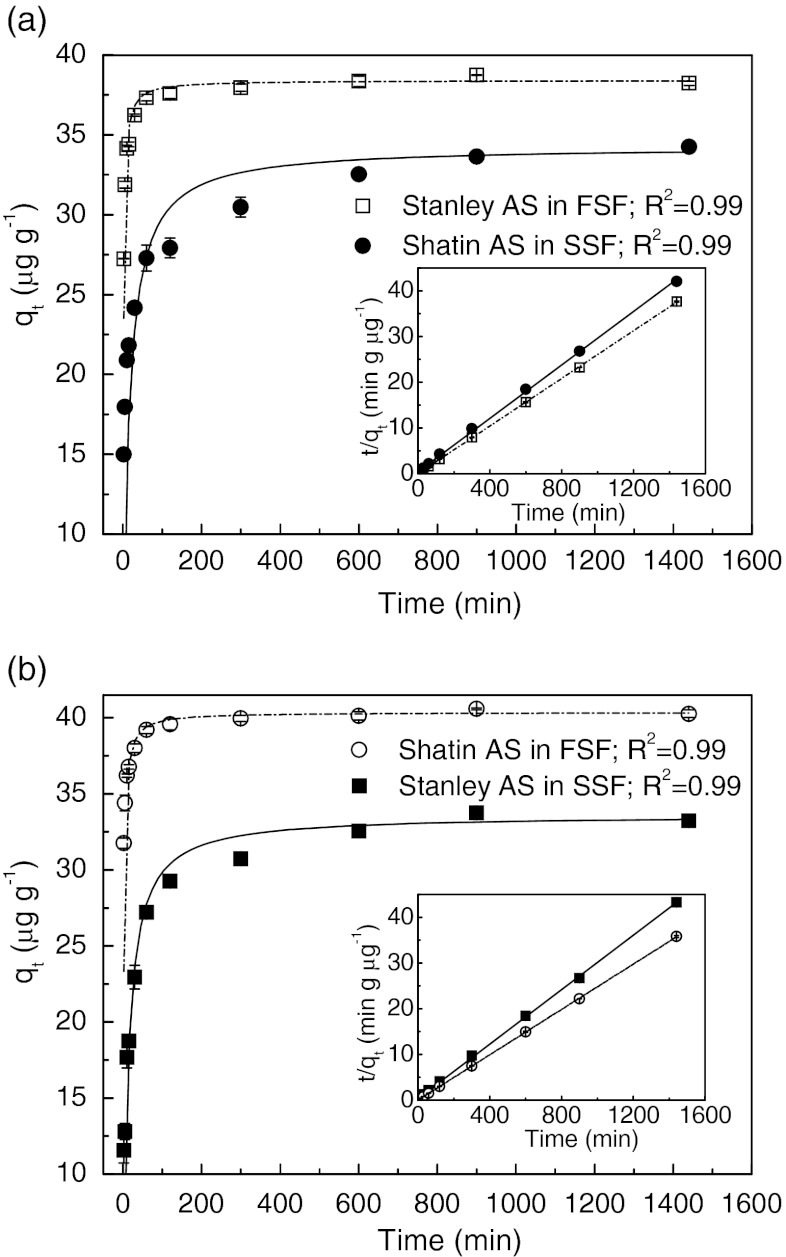
Adsorption kinetics (pseudo-second-order model) of tetracycline on AS. The *inset* is the linear plot of the pseudo-second-order model fit. *Error bars* mean SE. AS, activated sludge; FSF, freshwater sewage filtrate; SSF, saline sewage filtrate

Compared with the pseudo-first-order and Elovich models, the adsorption of tetracycline under all experimental conditions fitted the pseudo-second-order kinetic model best with R^2^ ≥ 0.99 (Table 1 and Table S3). This is in agreement with many other studies on the adsorption of tetracycline by various adsorbents, such as rectorite (Chang et al. 2009), calcined magnesium–aluminum hydrotalcites (Xu et al. 2009), and iron oxides–coated quartz (Tanis et al. 2008). As mentioned in “Adsorption kinetics,” tetracycline adsorption by Stanley AS in FSF (group I) and by Shatin AS in SSF (group II) reflected the actual adsorption behavior in the AS processes of Stanley and Shatin WWTPs. As shown in Fig. 1a, after a very rapid adsorption during the first 0.5 and 2 h, complete equilibriums were reached in 2 and 15 h for AS processes of Stanley and Shatin WWTPs, respectively. Judging from the pseudo-second-order rate constants (*k*_2_) of 2.04 × 10^−2^ and 1.94 × 10^−3^ g min^−1^ μg^−1^ as well as the initial rate ${{k}_2}q_{\mathrm{e}}^2$ of 30.1 and 2.28 μg min^−1^ g^−1^, it could be concluded that the adsorption of tetracycline in Stanley WWTPs was much faster than that in Shatin WWTPs. Additionally, greater *q*_e_ suggested greater adsorption capacity and higher removal efficiency of tetracycline when the same initial concentration (100 μg L^−1^) was applied in these two AS systems.

**Table 1:** Pseudo-second-order kinetic model parameters for tetracycline adsorption to activated sludge

Group	Adsorption system	*C*_0_ (μg L^−1^)	*q*_e_ (μg g^−1^)	$q_{\mathrm{e}}^{ *}\left( {\mu {\mathrm{g}}\;{{\mathrm{g}}^{{ - 1}}}} \right)$	*k*_2_ (g min^−1^ μg^−1^)	*R* ^2^
I	Stanley AS in FSF	99.2	38.4	38.8	2.04 × 10^−2^	0.99
II	Shatin AS in SSF	100.0	34.3	34.2	1.94 × 10^−3^	0.99
III	Stanley AS in SSF	98.7	33.6	33.7	2.35 × 10^−3^	0.99
IV	Shatin AS in FSF	104.3	40.3	40.6	1.70 × 10^−2^	0.99

*q*_*e*_ calculated values based on pseudo-second-order kinetics model fitting, $q_e^{ * }$ experimental values

To distinguish the key factors (AS properties and/or sewage matrix) affecting the above different adsorption behavior, another two sets of experiments with the different combinations of AS and sewage types, i.e., Stanley AS in SSF (group III) and Shatin AS in FSF (group IV), were conducted. As shown in Fig. 1 and Table 1, similar adsorption behaviors (*k*_2_ and *q*_e_) were observed for group I and group IV, which represented two types of AS in the same sewage matrix (FSF). For group II and group III, similar adsorption trends (*k*_2_ and *q*_e_) were also found in these two AS systems with SSF as the sewage matrix. However, comparing the groups using the same AS but different sewage matrixes, i.e., group I and group III or group II and group IV, the adsorption phenomenon varied greatly. Thus, these results suggested that the sewage matrix (FSF or SSF) rather than the AS played a determinant role in the adsorption of tetracycline on AS. To confirm the above conclusion, the characterization of activated sludge and sewage was further conducted, respectively.

#### Characteristics of AS

The characteristics of AS from Stanley and Shatin WWTPs, including zeta potential, particle size distribution, specific surface area, and RH were summarized in Table 2. AS has a negative surface charge, indicated by the negative value of zeta potential (Kara et al. 2008). The zeta potential of Stanley AS and Shatin AS was −22.5 and −12.5 mV, respectively. A significantly higher zeta potential was observed for Shatin AS, possibly due to the presence of abundant cations in the saline sewage (Table 2), especially the bivalent cations (Ca^2+^ and Mg^2+^), which may effectively lessen the negative surface charge of AS (Pevere et al. 2007). Both the mean and median size of Stanley AS were slightly smaller than those of Shatin AS while the specific surface areas of these two types of AS were almost the same. The RH is a suitable parameter to characterize the average hydrophobicity of the heterogeneous AS (Jin et al. 2003). In this study, Stanley AS and Shatin AS had RH values of 67 and 82 %, respectively, indicating the presence of both hydrophobic and hydrophilic groups at the sludge surface, and Shatin AS was slightly more hydrophobic than Stanley AS. Similar hydrophobicity results for AS (50–85 %) were reported by Jin et al. (2003) and Laurent et al. (2009).

**Table 2:** Characteristics of activated sludge and sewage from aeration tank

Property	Activated sludge	
	Stanley WWTP	Shatin WWTP
Zeta potential (mV)	−22.5 ± 1.4	−12.5 ± 0.7
Particle size distribution (μm)	Mean 106 ± 0	Mean 137 ± 0
	Median 80.9 ± 0.1	Median 116 ± 0
Specific surface area (m^2^ g SS^−1^)	28.2 ± 5.3	28.6 ± 4.5
RH (%)	67 ± 1	82 ± 0
Concentration (mg L^−1^)	Sewage from aeration tank	
	Freshwater sewage (Stanley WWTP)	Saline sewage (Shatin WWTP)
Na^+^	24.0 ± 0.3	3,497 ± 23
Ca^2+^	11.5 ± 0.2	116 ± 0
Mg^2+^	2.6 ± 0.1	301 ± 12
Cl^-^	37.5 ± 0.2	5,971 ± 10
SO_4_^2-^	24.9 ± 5.6	679 ± 14
DOC	4.2 ± 0.1	7.1 ± 0.1
Salinity (ppt)	0.1 ± 0.0	10.6 ± 0.0

#### Aqueous solution chemistry characteristics of sewage 

The concentration of the cations (Na^+^, Ca^2+^, and Mg^2+^), anions (Cl^−^ and SO_4_^2−^), DOC, and salinity of freshwater and saline sewage were also summarized in Table 2. Except for DOC, there was a vast difference for concentrations of cations, anions, and salinity between these two types of sewage. For Na^+^, Mg^2+^, Cl^−^, and salinity, the concentrations in the saline sewage were 106–160 times higher than those in the freshwater sewage, while for Ca^2+^ and SO_4_^2−^, the concentrations in the saline sewage were about 10 and 27 times higher than those in the freshwater sewage. The comparison of the ion concentration as well as salinity between the saline sewage and seawater indicated that seawater accounted for a fraction of about 30 % in the saline sewage.

#### Effect of ion species/concentration

**Fig. 2:**
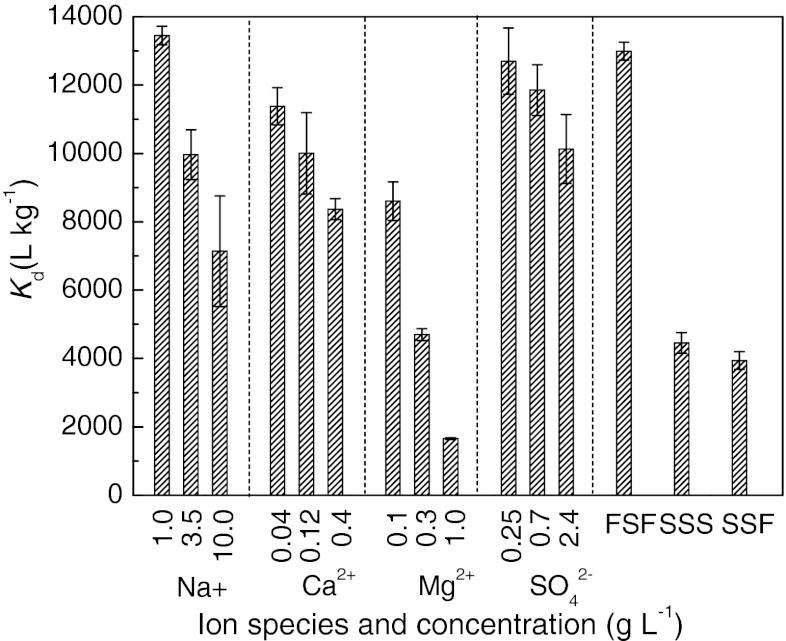
Effect of ion species and concentration on tetracycline adsorption. *Error bars* mean SE. FSF, freshwater sewage filtrate; SSS synthetic saline sewage; SSF, saline sewage filtrate

As mentioned above, the sewage matrix (FSF or SSF) rather than the AS played a determinant role in the different adsorption behavior of tetracycline on AS in freshwater and saline sewage systems, respectively. Adsorption distribution coefficient *K*_d_ was used to further examine the effect of the ion species and ion concentration on tetracycline adsorption (Sun et al. 2010; Ter Laak et al. 2006). As shown in Fig. 2, the *K*_d_ in FSF was 3.3 times as high as that in SSF while *K*_d_ in SSS was very similar to that in SSF. This indicated that the decrease of tetracycline adsorption in SSF might mainly result from the ions existed in SSF. This result is also in agreement with the previous study which reported that adsorption of tetracycline by marine sediment decreased with an increase of salinity (Xu and Li 2010).

However, salinity is a comprehensive parameter, and the previous studies did not specify the effect of each kind of ion on tetracycline adsorption. To distinguish the effects of different ions, NaCl, CaCl_2_, MgCl_2_, and Na_2_SO_4_ were respectively spiked into FSF at three concentration levels and their concentrations were calculated based on Na^+^, Ca^2+^, Mg^2+^, and SO_4_^2−^ as shown in Fig. 2. Judging from the *K*_d_ values at the middle concentration levels which were equal to their actual concentrations in SSF, it could be concluded that the decrease of adsorption in SSF compared to that in FSF was mainly due to the presence of Mg^2+^. The impact of Cl^−^ was negligible because the corresponding Cl^−^ concentration (1.5 g L^−1^) of 1.0 g L^−1^ Na^+^ was much higher than that (0.89 g L^−1^) of 0.3 g L^−1^ Mg^2+^ while the *K*_d_ (similar to the *K*_d_ in FSF) of the former was much greater than that of the latter. At the actual concentration level, the effect of Na^+^ on decreasing tetracycline adsorption was comparable with that of Ca^2+^ but much smaller than that of Mg^2+^ although the mole concentration ratios of ${{C}_{{{\mathrm{N}}{{\mathrm{a}}^{ + }}}}}/{{C}_{{{\mathrm{C}}{{\mathrm{a}}^{{2 + }}}}}}\;{\mathrm{and}}\;{{C}_{{{\mathrm{N}}{{\mathrm{a}}^{ + }}}}}/{{C}_{{{\mathrm{M}}{{\mathrm{g}}^{{2 + }}}}}}$ were greater than 50 and 12, respectively. This indicated that the effect of monovalent Na^+^ on adsorption was much less important than that of divalent Ca^2+^ and Mg^2+^, and nonspecific electrostatic interactions should not be the predominant adsorption mechanism of tetracycline on AS (Tanis et al. 2008).

Tetracycline may form strong complexes with Ca^2+^ and Mg^2+^, and the complexation of tetracycline with Ca^2+^ and Mg^2+^ in the aqueous phase might cause the decreased adsorption (Figueroa and Mackay 2005; Jin et al. 2007; Tanis et al. 2008). Additionally, the adsorption competition between positively charged quaternary ammonium functional group of tetracycline and divalent cations (Ca^2+^ and Mg^2+^) for the cation exchange sites of adsorbent surface (e.g., carboxyl groups on extracellular polymeric substance of AS) will also decrease tetracycline adsorption (Liu et al. 2010; Pils and Laird 2007; Sun et al. 2009; Sun et al. 2010; Tanis et al. 2008). Comparing the *K*_d_ values corresponding to Ca^2+^ of 0.4 g L^−1^ (0.01 M), Mg^2+^ of 0.1 g L^−1^ (0.004 M), and 0.3 g L^−1^ (0.0125 M), it can be found that the ability of Ca^2+^ to decrease tetracycline adsorption was weaker than that of Mg^2+^ given the same mole concentration. The *K*_d_ values at three different concentrations demonstrated that the adsorption decreased with the increase of ion concentration and the trend was the most significant for Mg^2+^. The occurrence of SO_4_^2−^ in SSF imposed a negligible impact on tetracycline adsorption.

#### Effect of pH

**Fig. 3:**
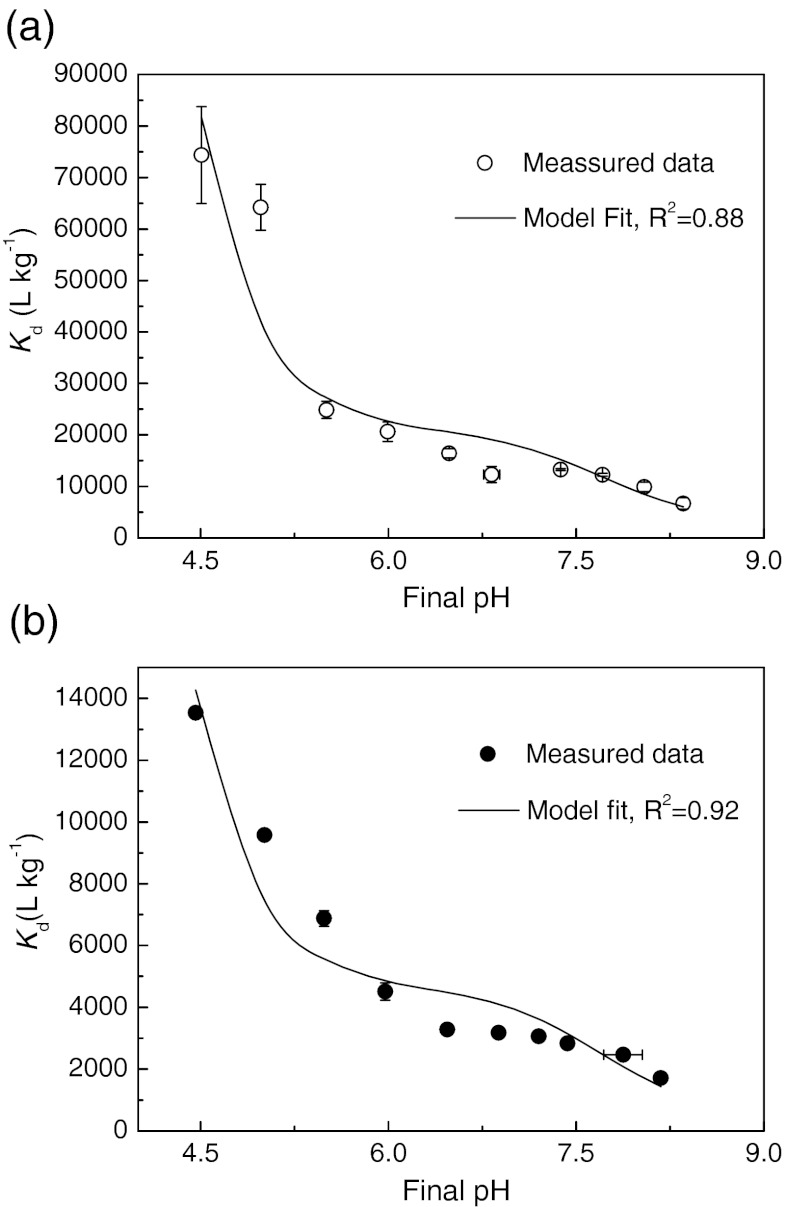
Effect of pH on *K*_d_ for tetracycline adsorption to AS: **a** Stanley AS in FSF and **b** Shatin AS in SSF. *Error bars* mean SE. AS, activated sludge; FSF, freshwater sewage filtrate; SSF, saline sewage filtrate

As shown in Fig. 3, *K*_d_ of tetracycline decreased with increasing pH over the tested pH range (4.5–8.4) and a gradual decrease trend was found between pH of 6.5 and 8.0 in both AS systems. Similar trend was also reported by other studies which investigated the pH effect on tetracycline adsorption to montmorillonite and soils (Figueroa et al. 2004; Sassman and Lee 2005). For Shatin AS system, the *K*_d_ decreases more than 7.5 times over the whole experimental pH range, from 13,530 ± 70 to 1,710 ± 30 L kg^−1^. For the Stanley AS system, the *K*_d_ decreased more significantly, from 74,340 ± 9,390 to 6,670 ± 1,100 L kg^−1^, by approximately 11 times over a similar pH range. As illustrated in Fig. S4 (see Electronic supplementary material), tetracycline possesses multiple ionizable functional groups, i.e., tricarbonyl amide (C-1/C-2/C-3), phenolic diketone (C-10/C-11/C-12), and dimethylamine (C-4) groups which correspond to three acid dissociation constants (p*K*_a_ = 3.3, 7.7, and 9.7), respectively. Thus, tetracycline exists as a cationic (+00), zwitterionic (+−0), and anionic (+−− or 0−−) species under acidic, moderately acidic to neutral, and alkaline conditions (Fig. S5, see Electronic supplementary material). It was reported that the ionization behavior is expected to significantly affect tetracycline sorption and each ionic species had its own adsorption magnitude (Figueroa et al. 2004; Gu and Karthikeyan 2005; Wang et al. 2008). To study the adsorption of different tetracycline species, the following empirical model, which expressed the overall adsorption distribution coefficient *K*_d_ as the sum of the species-specific adsorption distribution coefficients weighted with the corresponding fraction of individual species, was employed in this study.

8 $$ {{K}_{\mathrm{d}}} = K_{\mathrm{d}}^{{ + 00}} \times {{f}^{{ + 00}}} + K_{\mathrm{d}}^{{ + - 0}} \times {{f}^{{ + - 0}}} + K_{\mathrm{d}}^{{ + - - }} \times {{f}^{{ + - - }}} $$

where $K_{\mathrm{d}}^{{ + 00}},\;K_{\mathrm{d}}^{{ + - 0}},\;{\mathrm{and}}\;K_{\mathrm{d}}^{{ + - - }}$ are the species-specific adsorption distribution coefficients and *f* ^+00^, *f* ^+−0^, and *f* ^+−−^ are the fractions for cationic, zwitterionic, and anionic species, respectively. The anionic species (0−−) was neglected in the model due to its minor fraction in the tested pH range.

A nonlinear regression line was fitted to the *K*_d_ data at different pH values with Eq. (8) using Microsoft Office Excel 2003 software (Solver function). Well fits were obtained with the $K_{\mathrm{d}}^{{ + 00}},\;K_{\mathrm{d}}^{{ + - 0}},\;{\mathrm{and}}\;K_{\mathrm{d}}^{{ + - - }}$ of 1.06 × 10^6^, 2.10 × 10^4^, and 3.02 × 10^3^ L kg^−1^ in the Stanley AS system (*R* ^2^ = 0.88) while 1.54 × 10^5^, 4.62 × 10^3^, and 4.11 × 10^2^ L kg^−1^ in the Shatin AS system (*R* ^2^ = 0.92), respectively. This suggested that the adsorption affinity of different tetracycline species with AS surface followed the order of cationic >> zwitterionic species > anionic species. The above model, Eq. (8), was also used to fit tetracycline adsorption on soils (Sassman and Lee 2005) and montmorillonite (Figueroa et al. 2004). They found similar results indicating that cationic species had much higher affinity with adsorbents than zwitterionic species. The adsorption of anionic species was neglectable as $K_{\mathrm{d}}^{{ + - - }}$ was 0 (Sassman and Lee 2005). However, it was also reported that the adsorption of anionic tetracycline on montmorillonite was significant and $K_{\mathrm{d}}^{{ + - - }}$ was about half of $K_{\mathrm{d}}^{{ + - 0}}$ (Wang et al. 2008). By calculating the species-specific adsorption distribution coefficients weighted with the corresponding fraction, it was found that the contribution of zwitterionic tetracycline to the overall adsorption was always greater than 90 % in the practical pH range of aeration tank (6.0–7.0). In the lower pH range (6.0–4.5), although the fraction of cationic species was as low as 0.2–6.4 %, its contribution to the total adsorption ranged from 6.5 to 75 % in both Shatin and Stanley AS systems. In the higher pH range (7.7–8.4), the fraction of anionic species was about 50 to 80 % and its contribution to the total adsorption ranged 12–40 % in these two AS systems.

#### Adsorption isotherms

Adsorption isotherms were utilized to assess tetracycline distributions between AS and aqueous phases as a function of tetracycline concentration. Freundlich and Langmuir adsorption isotherms were applied to fit the experimental data at three temperatures (10, 25, and 35 °C):

9 $$ \begin{array}{*{20}c} {{\mathrm{Freundlich}}\;{\mathrm{model}}:} & {{{q}_{\mathrm{e}}} = {{K}_{\mathrm{f}}}C_{\mathrm{e}}^n} \\ \end{array} $$

10 $$ \begin{array}{*{20}c} {{\mathrm{Langmuir}}\;{\mathrm{model}}:} & {{{q}_{\mathrm{e}}} = \frac{{{{q}_{{{\max }}}}b{{C}_{\mathrm{e}}}}}{{1 + b{{C}_{\mathrm{e}}}}}} \\ \end{array} $$

where *K*_f_ (in micrograms^1 − *n*^ liter^*n*^ per gram) is the Freundlich affinity coefficient and gives an estimate of the adsorptive capacity, *n* (unitless) is the Freundlich linearity index, *q*_max_ (in micrograms per gram) is the maximum adsorption capacity, and *b* (in liters per microgram) is the Langmuir equilibrium coefficient.

**Fig. 4:**
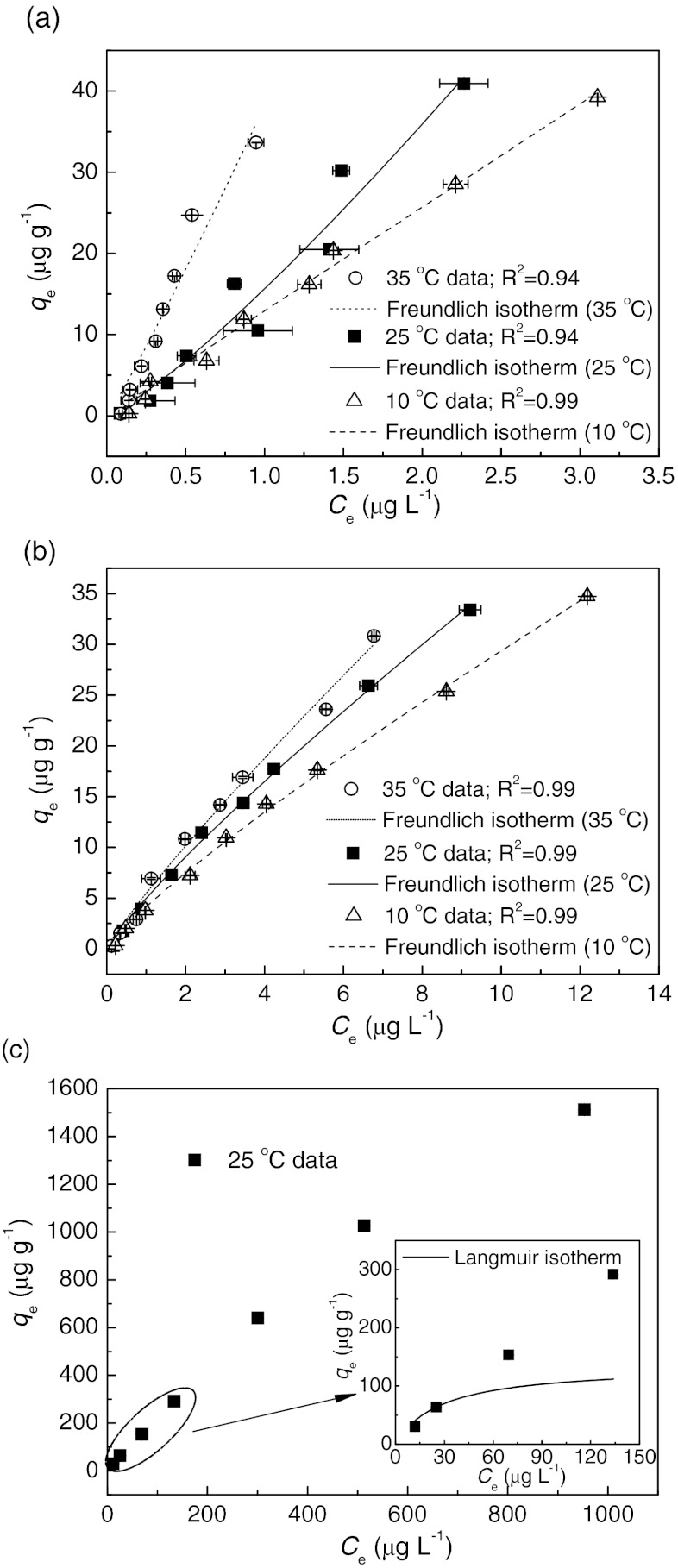
Adsorption isotherms of tetracycline on AS at 10, 25, and 35 °C: **a** Stanley AS in FSF (initial concentration 1–100 μg L^−1^), **b** Shatin AS in SSF (initial concentration 1–100 μg L^−1^), and **c** Shatin AS in SSF (initial concentration 100–5,000 μg L^−1^). *Error bars* mean SE. AS, activated sludge; FSF, freshwater sewage filtrate; SSF, saline sewage filtrate

The model fitting parameters were summarized in Table S4 (see Electronic supplementary material). Judging from the correlation coefficients *R* ^2^, it seems that the fitting performance of the Langmuir model was as good as that of the Freundlich model. However, the Freundlich rather than the Langmuir model was preferred as the applicable adsorption isotherm to fit the experimental data in this study due to the following reasons: Firstly, significantly inconsistent *q*_max_ and *b* values of the Langmuir model were obtained for Stanley AS system at different temperatures. For example, the *q*_max_ at 25 °C was 2 orders of magnitude greater than that at 10 °C, and this is unreasonable because temperature will not alter the maximum adsorption capacity of AS so much. Secondly, the predicted value of *q*_max_ based on the Langmuir model was not consistent with the actual situation. Taking Shatin AS system for example, the *q*_max_ of 135 μg g^−1^ at 25 °C suggested that the saturate adsorption amount of tetracycline on AS was 135 μg g^−1^ and it should not increase further even when the initial tetracycline concentration increased. However, as shown in Fig. 4c, the adsorption did not reach saturation at 135 μg g^−1^ and *q*_e_ raised from 30.4 to 1510 μg g^−1^ with the initial tetracycline concentration increasing from 100 to 5,000 μg L^−1^. The possible reason might be that the basic assumption of the Langmuir adsorption model did not stand for the tested AS, that is, the adsorption of tetracycline on AS was multilayer instead of monolayer. Therefore, the Freundlich isotherm was applied to describe the adsorption of tetracycline on AS. In previous studies, the adsorption of tetracycline on soils (Wan et al. 2010), marine sediment (Xu and Li 2010), carbon nanotubes (Ji et al. 2010), and montmorillonite (Wang et al. 2008) was also found to follow the Freundlich isotherm better than the Langmuir isotherms.

Freundlich adsorption isotherm fittings at three temperatures for Stanley AS and Shatin AS were presented in Fig. 4a, b, respectively. Visually, higher temperature could enhance the adsorption process and the similar result was reported before (Tanis et al. 2008). In addition, *K*_f_ can be also regarded as a relative indicator of adsorption capacity, i.e., greater *K*_f_ indicated higher adsorption capacity of the adsorbent (Xu and Li 2010). When temperature increased from 10 to 35 °C, *K*_f_ raised from 12.9 to 38.2 (in micrograms^1 − *n*^ liter^*n*^ per gram) and from 4.15 to 5.48 (in micrograms^1 − *n*^ liter^*n*^ per gram) in these two adsorption systems, respectively. At the same temperature, the *K*_f_ of Stanley AS was approximately three times (10 and 25 °C) and seven times (35 °C) greater than the corresponding *K*_f_ of Shatin AS.

## Conclusions

The removal mechanisms and kinetics of trace tetracycline, which were affected by aqueous solution chemistry properties of sewage and activated sludge surface characteristics, were systematically investigated in this study. The major conclusions were as follows:

- Adsorption is the primary removal mechanism for tetracycline in both freshwater and saline sewage activated sludge systems while biodegradation, volatilization, and hydrolysis can be completely ignored. This will enhance the transport of tetracycline into soil environment and increase the risk of development/maintenance/transfer/spread of tetracycline-resistant bacteria and tetracycline-resistant genes in the long term.
- Adsorption of tetracycline on AS fitted pseudo-second-order kinetics model well with R^2^ ≥ 0.99. Faster adsorption rate (*k*_2_ 2.04 × 10^−2^ g min^−1^ μg^−1^) and greater adsorption capacity (*q*_e_ 38.8 μg g^-1^) were found for freshwater AS than saline AS (*k*_2_ 1.94 × 10^−3^ g min^−1^ μg^−1^, *q*_e_ 34.3 μg g^−1^).
- The Mg^2+^ in the saline sewage played a predominant role in decreasing tetracycline adsorption on saline AS.
- Adsorption of tetracycline decreased significantly with increasing pH over the tested pH range (4.5–8.4) and *K*_d_ was reduced from 13,530 ± 70 to 1,710 ± 30 L kg^−1^ in saline AS system and from 74,340 ± 9,390 to 6,670 ± 1,100 L kg^−1^ in freshwater AS system. The adsorption affinity of different tetracycline species with AS surface followed the order of cationic species >> zwitterionic species > anionic species. Contribution of zwitterionic tetracycline to the overall adsorption was always greater than 90 % over the actual pH range (6.0–7.0) in aeration tank.
- Adsorption of tetracycline in a wide range of temperature (10 to 35 °C) followed the Freundlich adsorption isotherm well with *R* ^2^ ranging from 0.94 to 0.99.

## Electronic supplementary material

Below is the link to the electronic supplementary material.

ESM 1 (DOC 594 kb)
