# Supplementary material for: Removal mechanisms and kinetics of trace tetracycline by two types of activated sludge treating freshwater sewage and saline sewage
Source: Environ Sci Pollut Res Int. 2012 Oct 2;20(5):3024–33. doi: 10.1007/s11356-012-1213-5 (PMC3633786; doi:10.1007/s11356-012-1213-5)
Supplement: Supplementary file 1 — (DOC 594 kb) [file 11356_2012_1213_MOESM1_ESM.doc]

**ELECTRONIC SUPPLEMENTARY MATERIAL for**

Removal Mechanisms and Kinetics of Trace Tetracycline by Two Types of Activated Sludge Treating Freshwater Sewage and Saline Sewage

Bing Li and Tong Zhang^*^

Environmental Biotechnology Laboratory, Department of Civil Engineering,
The University of Hong Kong, Hong Kong SAR

Submitted to *Environmental Science and Pollution Research*

*Corresponding author phone: +852-28578551; fax: +852-25595337; e-mail: zhangt@hkucc.hku.hk

**S 2.2 *Removal of tetracycline in activated sludge process***

**S 2.5 UPLC/MS/MS Analysis**

**Tables**

Table S1. Operation parameters and treatment processes of Shatin and Stanley WWTPs

Table S2. Batch experimental design

Table S3. Pseudo-first-order kinetics and Elovich model parameters for tetracycline adsorption to activated sludge

Table S4. Freundlich and Langmuir isotherm parameters for tetracycline adsorption to activated sludge

**Figures**

Fig. S1. Removal of tetracycline in (a) freshwater sewage system and (b) saline sewage system B: biodegradation; A: adsorption; V: volatilization; H: hydrolysis

Fig. S2. The impact of antibiotics on the activated sludge activity. (a) freshwater sewage system (b) saline sewage system.

Fig. S3. The inhibition effect of NaN_3_ on activated sludge biodegradation activity. (a) freshwater sewage system (b) saline sewage system.

Fig. S4**.** Chemical structure of tetracycline. The regions framed by dashed lines represent the structural moieties associated with the three acidic dissociation constants ($\text{p}K_{\text{aS}}$)

Fig. S5**.** Distribution of different tetracycline species

**Total Pages: 12**

**Tables: 4**

**Figures: 5**

***S 2.2 Removal of tetracycline in activated sludge process***

For Treatment I, R1 and R1’ were duplicate reactors in which all four removal mechanisms, including biodegradation, adsorption, volatilization and hydrolysis occurred. In Treatment II (R2 and R2’), biodegradation was excluded because the activated sludge was inhibited by NaN_3_. In Treatment III (R3), tetracycline was eliminated via volatilization and hydrolysis due to the absence of activated sludge. Treatment IV (R4) and Treatment Control (R5) were utilized to examine the hydrolysis of tetracycline and the effect of tetracycline on the sludge bioactivity, respectively. In order to avoid possible photolysis, all reactors were placed in a closed chamber and tetracycline stock solution was spiked to obtain the initial concentration of 100 μg L^-1^. The aeration and mixing were supplied by the magnetic stirrers at 100 rpm. Slurry samples were taken from the batch reactors at the following times: 0, 0.25, 0.5, 1, 2, 5, 10, 15, 24h, respectively. Comparison of the caffeine biodegradation profile in Treatment Control with that in Treatment I (Fig. S2) indicated that the impact of tetracycline on the sludge biological activity was negligible. In addition, caffeine concentration kept constant in Treatment II during 24 h while it was completely biodegraded within 10 h in Treatment I, suggesting that the biological activity of sludge in Treatment II was thoroughly inhibited by NaN_3_ (Fig. S3).

***S 2.5 UPLC/MS/MS analysis***

About 5 mL sample was withdrawn from conical flasks using glass syringe and filtered via 0.2 µm cellulose nitrate membrane which showed no adsorption of tetracycline [1] The first 2 mL filtrate was discarded and the following 1.0 mL was collected in an amber vial. The initial mobile phase proportion, 95% (H_2_O) : 5% (ACN), was kept for 1.5 min. Then H_2_O was linearly decreased to 40% within 2.5 min. After that, H_2_O was drastically decreased to 0% within 0.1 min and held for 0.8 min to clean the column using 100% ACN. At last, H_2_O was changed to the initial percentage (95%) in 0.1 min and kept for 1.0 min to equilibrate the column before the next injection. The total run time was 6.0 min and retention time of tetracycline was 3.04 min.

Table S1. Operation parameters and treatment processes of Shatin and Stanley WWTPs

| WWTPs | Inhabitant served  (× 10^3^) | Average daily flows (m^3^) | HRT  (h) | SRT  (d) | Sewage type | Major treatment processes |
| --- | --- | --- | --- | --- | --- | --- |
| Shatin | 600 | 2.3$\times$10^5^ | 10 | 12 | Saline sewage | Anoxic-Aerobic AS process |
| Stanley | 27 | 8.8$\times$10^3^ | 17 | 7 | Freshwater sewage | Anoxic-Aerobic AS process + Chlorination |

HRT: hydraulic retention time;

SRT: solid retention time

Table S2. Batch experimental design

| Treatment Group | Reactor No. | Activated  Sludge | Wastewater | Antibiotics  (100 µg/L) | Caffeine^1^  (100 µg/L) | 1.0%^2^ NaN_3_ | Aeration | Removal routes |
| --- | --- | --- | --- | --- | --- | --- | --- | --- |
| I | R1 & R1’ | +^5^ | + | + | + | - | + | B+A+V+H^3^ |
| II | R2 & R2’ | + | + | + | + | + | + | A+V+H |
| III | R3 | -^6^ | + | + | + | + | + | V+H |
| IV | R4 | - | + | + | + | + | - | H |
| Control^4^ | R5 | + | + | - | + | - | + | - |

*1: Caffeine was used as the reference organic chemical to indicate biological activity of sludge (Kim et al., 2008) as caffeine is known to be*

*readily biodegradable and has no measurable adsorption to sludge.*

*2: NaN_3_ was used to inhibit the sludge biodegradation activity;*

*3: B-biodegradation; A-adsorption; V-volatilization; H-hydrolysis*

*4: Control was used to monitor the effect of antibiotics on the sludge activity*

*5: “+” indicated “with” or “presence”*

*6: “–” indicated “without” or “absence”*

Table S3. Pseudo-first-order kinetics and Elovich model parameters for tetracycline adsorption to activated sludge

| Group | Adsorption System | $C_{0}$ (μg L^-1^) | $q_{\text{e}}^{*}$ (μg g^-1^) | Pseudo-first-order kinetics | | | Elovich model | | |
| --- | --- | --- | --- | --- | --- | --- | --- | --- | --- |
|  |  |  |  | $q_{\text{e}}$(μg g^-1^) | $k_{1}$(1 min^-1^) | $R^{2}$ | $a$ | $b$ | $R^{2}$ |
| I | Stanley AS in FSF | 99.2 | 38.8 | 3.13 | 1.84×10^-3^ | 0.49 | 1.42 | 29.7 | 0.80 |
| II | Shatin AS in SSF | 100.0 | 34.2 | 12.6 | 3.45×10^-3^ | 0.95 | 2.94 | 13.8 | 0.99 |
| III | Stanley AS in SSF | 98.7 | 33.7 | 11.6 | 2.53×10^-3^ | 0.81 | 3.64 | 9.43 | 0.96 |
| IV | Shatin AS in FSF | 104.3 | 40.6 | 2.90 | 1.84×10^-3^ | 0.58 | 1.19 | 32.9 | 0.86 |

$q_{\text{e}}$: calculated values based on pseudo-first-order kinetics model fitting;

$q_{\text{e}}^{*}$: experimental values;

AS: activated sludge;

FSF: freshwater sewage filtrate;

SSF: saline sewage filtrate

Table S4. Freundlich and Langmuir isotherm parameters for tetracycline adsorption to activated sludge

| Adsorption System | Temperature  (°C) | Freundlich | | | Langmuir | | |
| --- | --- | --- | --- | --- | --- | --- | --- |
|  |  | $K_{\text{f}}$ (μg^1-^*^n^* L*^n^* g^-1^) | $n$ | $R^{2}$ | $q_{\text{max}}$(μg g^-1^) | $b$(L μg^-1^) | $R^{2}$ |
| Stanley AS in FSF | 10 | 12.9 | 0.991 | 0.99 | 8.29×10^2^ | 1.61×10^-2^ | 0.99 |
|  | 25 | 15.6 | 1.20 | 0.94 | 8.71×10^4^ | 1.97×10^-4^ | 0.93 |
|  | 35 | 38.2 | 1.08 | 0.94 | 2.01×10^4^ | 1.84×10^-3^ | 0.93 |
| Shatin AS in SSF | 10 | 4.15 | 0.850 | 0.99 | 1.32×10^2^ | 2.88×10^-2^ | 0.99 |
|  | 25 | 4.99 | 0.862 | 0.99 | 1.35×10^2^ | 3.56×10^-2^ | 0.99 |
|  | 35 | 5.48 | 0.888 | 0.99 | 1.53×10^2^ | 3.56×10^-2^ | 0.99 |

Fig.S1 Removal of tetracycline in (a) freshwater sewage system and (b) saline sewage system B: biodegradation; A: adsorption; V: volatilization; H: hydrolysis

Fig. S2. The impact of antibiotics on the activated sludge activity. (a) freshwater sewage system and (b) saline sewage system.

Fig. S3. The inhibition effect of NaN_3_ on activated sludge biodegradation activity. (a) freshwater sewage system and (b) saline sewage system.


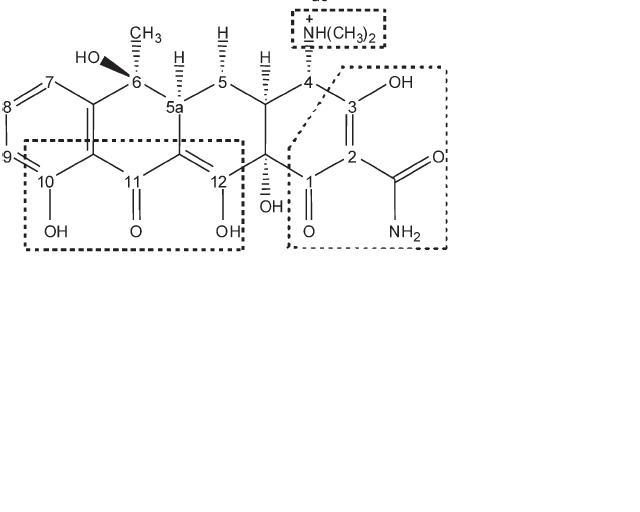

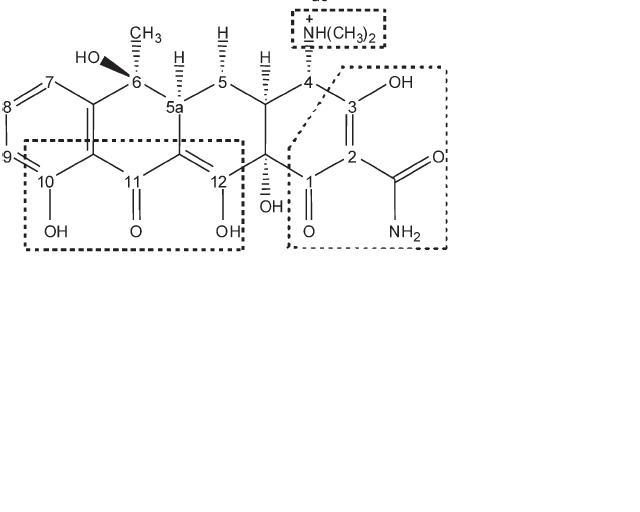


Fig. S4. Chemical structure of tetracycline. The regions framed by dashed lines represent the structural moieties associated with the three acidic dissociation constants ($\text{p}K_{\text{aS}}$) (Source：Sun et al., 2010 [2]; Wan et al., 2010 [3])

Fig. S5. Distribution of different tetracycline species (Source: Gu and Karthikeyan, 2005 [4])

**Reference**

[1] B. Li, T. Zhang, Z.Y. Xu, H.H.P. Fang, Rapid analysis of 21 antibiotics of multiple classes in municipal wastewater using ultra performance liquid chromatography-tandem mass spectrometry. Anal. Chim. Acta 645 (2009) 64-72.

[2] H.Y. Sun, X. Shi, J.D. Mao, D.Q. Zhu, Tetracycline sorption to coal and soil humic acids: an examination of humic structural heterogeneity. Environ. Toxicol. Chem. 29 (2010) 1934-1942.

[3] Y. Wan, Y.Y. Bao, Q.X. Zhou, Simultaneous adsorption and desorption of cadmium and tetracycline on cinnamon soil. Chemosphere 80 (2010) 807-812.

[4] C. Gu, K.G. Karthikeyan, Interaction of tetracycline with aluminum and iron hydrous oxides. Environ. Sci. Technol. 39 (2005) 2660-2667.
